# Bioactivity Evaluation of Biphasic Hydroxyapatite Bone Substitutes Immersed and Grown with Supersaturated Calcium Phosphate Solution

**DOI:** 10.3390/ma14185143

**Published:** 2021-09-08

**Authors:** Yusuke Yamaguchi, Tomonori Matsuno, Atsuko Miyazawa, Yoshiya Hashimoto, Takafumi Satomi

**Affiliations:** 1 Department of Oral and Maxillofacial Surgery, School of Life Dentistry at Tokyo, The Nippon Dental University, 1-9-20 Fujimi, Chiyoda-ku, Tokyo 102-8159, Japan; ygucci@tky.ndu.ac.jp (Y.Y.); atsuko-m@tky.ndu.ac.jp (A.M.); tsatomi@tky.ndu.ac.jp (T.S.); 2 Oral and Maxillofacial Surgery, The Nippon Dental University Hospital, 2-3-16 Fujimi, Chiyoda-ku, Tokyo 102-8158, Japan; 3 Department of Biomaterials, Osaka Dental University, 8-1, Kuzuhahanazonocho, Hirakata 573-1121, Osaka, Japan; yoshiya@cc.osaka-dent.ac.jp

**Keywords:** biphasic hydroxyapatite, low crystalline hydroxyapatite, supersaturated calcium phosphate solution

## Abstract

Recently, the frequency of use of bone substitute materials for the purpose of bone augmentation has increased in implant treatment, but bone formation with bone substitute materials alone is limited. Calcification of bone in the body progresses as Ca^2+^, H_2_PO^4-^, and HPO_4_^2-^ in the body form hydroxyapatite (HA) crystals. In this study, therefore, we prepared a biphasic bone substitute with biological activity to promote bone formation by inducing precipitation and growth of HA crystals on the surface of a bone substitute and evaluated it. Biphasic bone substitute granules were prepared by immersing HA granules in a supersaturated calcium phosphate solution prepared by mixing five medical infusion solutions, the precipitate was analyzed, and the biological activities of biphasic HA granules were evaluated in vitro and in vivo. As a result, the precipitated calcium phosphate crystals were identified as low crystalline HA. On the surface of the HA granules, low-crystalline HA grew markedly as needle-shaped crystals and significantly promoted cell proliferation and bone differentiation. In animal experiments, biphasic HA granules had a significantly higher bone mineral density, new bone volume ratio, and new bone area ratio. Therefore, it suggests that biphasic hydroxyapatite is a useful bone substitute for bone augmentation in dental implant treatment.

## 1. Introduction

Dental implant therapy combined with bone augmentation is becoming prevalent [1,2,3]. Bone graft materials used for bone augmentation include autologous bone, allogeneic bone, xenogeneic bone, and synthetic apatite, but autologous bone is considered to be the best bone graft material because it has osteogenic, osteoinductive, and osteoconductive properties [4]. However, there are problems such as the necessity of new surgical invasion for harvesting autologous bone with associated pain and complications including postoperative infection as well as the limited availability of autologous bone and bone resorption after grafting [5,6,7,8]. For these reasons, various bone substitutes have been developed and marketed. In dental implant therapy, allogenic bone, calcined bovine bone, or various synthetic materials such as hydroxyapatite (HA) and β-tricalcium phosphate (β-TCP) have been used clinically.

In the body, calcification occurs as HA crystals formed from Ca, P, and hydroxide ions are deposited on collagen fibers. Moreover, crystallization of HA progresses as Ca^2+^, H_2_PO^4−^, and HPO_4_^2−^ form the base of HA crystals due to the interaction of phospholipids in matrix vesicles, are ionically bonded in matrix vesicles, and grow as mineralized nodules through repeated precipitation; this mechanism is involved in bone calcification [9]. HA is a major inorganic component of human bones and teeth. A large number of studies of synthetic HA have clarified its biological behavior in the body, and it has been shown to have excellent biocompatibility, have biological activities and osteoconductivity to promote bone growth, and directly bind to bone without mediation by fibrous tissue in bone [10]. For these reasons, synthetic HA has been used clinically as a bone substitute for repair of bone defects for more than 25 years [11]. However, new bone formation has been limited with HA alone because of the lack of osteogenicity and osteoinductivity.

To reproduce calcification in the body, i.e., apatite formation, in vitro, simulated body fluid (SBF) with ion concentrations and pH equal to those in human plasma was developed [12]. When bioactive glass (Na_2_O–CaO–SiO_2_–P_2_O_5_), which has been applied to basic research and clinical practice as a ceramic bioactive material for a long time is immersed in SBF, an apatite layer consisting of Ca and P contained in SBF is formed on the surface of the material [13,14,15,16]. Therefore, SBF has been used for in vitro evaluation of in vivo biological activities of bone and bone-bonding ability [17]. However, as the amounts of Ca and P in SBF are fixed, the amount of apatite that precipitates is limited. However, Oyane et al. [18]. developed a technique to form an apatite layer on the surface of materials using a supersaturated calcium phosphate solution prepared using amorphous calcium phosphate, which is an apatite precursor, by mixing common medical infusion solutions, such as dipotassium phosphate and Ringer’s solutions, and adjusting the Ca/P ratio close to 1.67. In addition, Mutsuzaki et al. reported that bone-fixation titanium screws can be more firmly fixed to bone by immersing them in a supersaturated calcium phosphate solution obtained by mixing five medical infusion solutions [19]. Furthermore, in recent years, clinical studies have been conducted to show the safety and efficacy of supersaturated calcium phosphate solutions [20].

Therefore, we considered that an apatite layer that promotes bone regeneration could be efficiently precipitated in a short time, and focused on a supersaturated calcium phosphate solution. In this study, we prepared biphasic HA granules by having an apatite layer deposited on the surface or in the pores of HA granules and evaluated their biological activities to promote new bone formation in vitro and in vivo.

## 2. Materials and Methods

### 2.1. Preparation of Supersaturated Calcium Phosphate Solution

Supersaturated calcium phosphate solution was prepared according to the report of Mutsuzaki et al. [19] by mixing five medical infusion solutions. Two calcium-containing solutions (4.5 mM Ca^2+^), i.e., calcium chloride corrective injection (Otsuka Pharmaceutical Co., Ltd., Tokyo, Japan) and Ringer’s solution (Otsuka Pharmaceutical Co., Ltd., Tokyo, Japan), were mixed, two phosphorus-containing solutions (20 mM PO_4_^3−^), i.e., sodium phosphate corrective solution (Otsuka Pharmaceutical Co., Ltd., Tokyo, Japan) and Klinisalz^®^ infusion solution (Otsuka Pharmaceutical Co., Ltd., Tokyo, Japan), were mixed, and Meylon^®^ 7% injection solution, which is a sodium hydrogen carbonate injection solution for alkalinization (Otsuka Pharmaceutical Co., Ltd., Tokyo, Japan), was added. Table 1 shows the composition of each medical infusion solution. The supersaturated calcium phosphate solution was prepared by mixing these five medical infusion solutions at a Ca/P ratio of 2.0 and a pH of 7.8 at 37 °C.

### 2.2. Determination of Ca and P Concentrations in Supernatants of Supersaturated Calcium Phosphate Solution by Absorbance Measurement

The Ca concentrations in the supernatants collected 15 min and 24, 48, and 72 h after preparation of the supersaturated calcium phosphate solution were determined by measuring the absorbance at 610 nm using a calcium kit (Calcium E-HA Test Wako: FUJIFILM Wako Pure Chemical Co., Osaka, Japan), and the phosphate concentrations were determined with a phosphate kit (Piblue Phosphsate Assay Kit: Funakoshi Co., Ltd., Tokyo, Japan) using a microplate reader (Bio-Rad Model 680; Bio-Rad Laboratories, Inc., Hercules, CA, USA) by measuring the absorbance at 620 nm (n = 4).

### 2.3. Morphological Observation of Precipitates

The surface properties of the precipitates that deposited after preparation of the supersaturated calcium phosphate solution were observed serially under a scanning electron microscope (SEM: S-900, Hitachi High-Tech Co., Tokyo, Japan).

### 2.4. X-ray Diffraction Analysis and Fourier Transform Infrared Spectrophotometry of HA Granules and Precipitates

HA (APACERAM-AX^®^: HOYA Technosurgical, Tokyo, Japan) granules and precipitates were collected 72 h after preparation of the supersaturated calcium phosphate solution, allowed to dry, and the crystallinity was analyzed by X-ray diffraction analysis (X-ray diffraction: XRD, XRD-6100, Shimadzu, Kyoto, Japan). In addition, the molecular structure was determined by Fourier transform infrared spectroscopy (FT–IR, IRAffinity-1S, Shimadzu, Kyoto, Japan) for qualitative analysis and identification of the precipitates.

### 2.5. Preparation of Biphasic Bone Substitute Granules

The bone substitute granules used in this study were HA. Biphasic HA granules were prepared by immersing HA granules (0.1 g) in 3.0 mL of the supersaturated calcium phosphate solution for 24, 48, and 72 h and leaving them to stand in an incubator at 37 °C.

### 2.6. Observation of Biphasic Bone Substitute Granules

The morphology of biphasic HA granules was examined under a stereoscopic microscope (Axio Zoom, Carl Zeiss Microscopy Ltd., Cambridge, England), and their surface properties were observed under SEM.

### 2.7. Cell Culturing

The osteoblast-like cell strain MC3T3-E1 (Riken cell bank, Tsukuba, Japan) was cultured in the culture media α-MEM (Nacalai tesque INC., Kyoto, Japan) supplemented with 10% Fetal bovine serum (FBS; Biowest, Nuaillé, France) and 1% penicillin-streptomycin (Life Technologies Japan Ltd., Tokyo, Japan) at 37 °C in a 5% CO_2_ atmosphere in a 75 cm^2^ flask (Corning Inc., St Lawrense, NY, USA). The culture media were renewed every three days.

### 2.8. Cytotoxicity Testing

A total of 500 μL of α-MEM medium retained MC3T3-E1 cells (5.0 × 10^4^ cell/well) were seeded on a 24-well plate (Cosmo Bio Co., Ltd., Tokyo, Japan), and 2.0 mL of α-MEM medium was added. Then, HA granules and biphasic HA granules (0.05 g each) were placed in an Intercell (Cosmo Bio Co., Ltd., Tokyo, Japan), and cell culturing was initiated. The culture medium was renewed every three days, and the cytotoxicity of biphasic HA granules was evaluated according to cell proliferation after one, three, five, and seven days with a MTT Cell Viability Assay Kit (Biotium Inc., Fremont, CA, USA) using a microplate reader by measuring the absorbance at 590 nm (n = 4).

### 2.9. Cell Differentiation Testing

Three days after seeding of MC3T3-E1 (1.0 × 10^3^ cells/mL) to a 24-well plate, the cells were transferred from the culture medium to a bone differentiation medium, which was changed to α-MEM with 10% FBS supplemented with 10 mM β-glycerophosphate (FUJIFILM Wako Pure Chemical Co., Osaka, Japan), 2.0 mM L-ascorbic acid 2-phosphate (FUJIFILM Wako Pure Chemical Co., Osaka, Japan), and 100 nM dexamethasone (FUJIFILM Wako Pure Chemical Co., Osaka, Japan). Similar to the cytotoxicity testing, HA granules and biphasic HA granules (0.05 g each) were placed in an Intercell inside a 24-well plate, incubated at 37 °C, and their bone differentiation abilities after seven days were evaluated with an ALP Assay kit (FUJIFILM Wako Pure Chemical Co., Osaka, Japan) using a microplate reader by measuring the absorbance at 470 nm (n = 4).

### 2.10. Animal Experiment

The animal experiment was performed at the Research Center for Odontology, Nippon Dental University School of Life Dentistry, in compliance with the Rules for Animal Experiments, Nippon Dental University School of Life Dentistry (approval number: 19-20-2).

**Figure 1:**
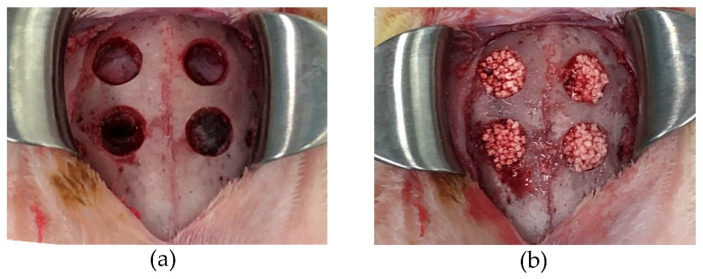
Rabbit cranium before and after filling of bone substitute granules; (**a**) before filling, (**b**) after filling.

Five 20-week-old male New Zealand white rabbits were used for the experiment. General anesthesia was applied by intramuscular injection of a mixture of butorphanol tartrate (Meiji Seika Pharma Co., Ltd., Tokyo, Japan), medetomidine hydrochloride (Nippon Zenyaku Kogyo Co., Ltd., Fukushima Japan), and midazolam (Astellas Pharma Inc, Tokyo, Japan). After confirming the effect of anesthesia, the parietal region was shaved and disinfected with 70% ethanol, 1.0 mL of 2% xylocaine (Dentsply Sirona Inc, Tokyo, Japan) containing epinephrine was applied for infiltration anesthesia, the scalp was incised with a No.15 scalpel, and the cranium was exposed by detaching the periosteum. Ensuring no damage to the dura mater of the brain, after forming a groove with a diameter of six mm using a bone trephin bar (Technika co., Ltd., Tokyo, Japan), a full-thickness bone defect was formed in four holes with an ultrasonic cutting tool (PIEZO SURGERY^®^; Mectron spa, Carasco, Italy) (Figure 1a). The bone defects were filled with 0.3 g of biphasic HA granules and HA granules as a control (Figure 1b). Two holes in one rabbit were left unfilled as a negative control.

The periosteum was given buried suture using 5-0 VICRIL^®^ (Ethicon Inc., Johnson & Johnson, Somerville, NJ, USA), and the skin was closed by suturing with a 5-0 nylon suture. Four weeks after surgery, the rabbits were euthanized by inhalation of high concentration CO_2_ in an airtight container. After sacrifice, the cranium was collected, fixed with 70% ethanol, trimmed, scanned by micro-CT (TRI/3D-BON; Ratoc System Engineering, Tokyo, Japan), and the state of new bone formation was compared.

**Figure 2:**
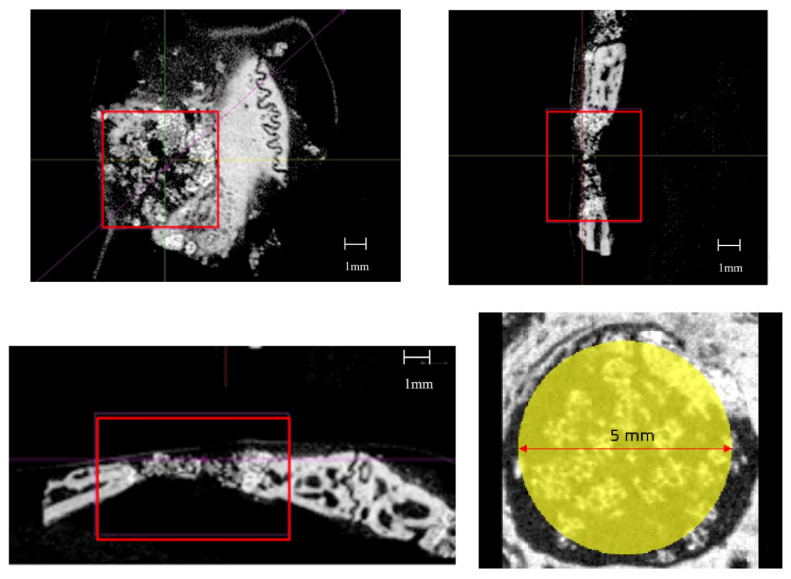
Micro–CT images and ROI.

For the measurement in microCT images, the full thickness of the cranium in an area 5 mm in diameter around the center of the defect was defined as the region of interest (ROI) (Figure 2). The volumes of newly formed bone and HA, volume of newly formed bone alone, and volume ratio of newly formed bone in ROI were compared between the biphasic HA and HA groups. The extraction threshold for newly formed bone was ≥2615 mg/cm^3^, and that for HA granules was ≥3,539 mg/cm^3^. Furthermore, bone mineral density (BMD) was compared in BMD images. Then, undecalcified, thickness of 30–40 μm decalcified polished samples were prepared by resin embedding and polymerization, and the area ratio of newly formed bone was compared in Villanueva bone stain images.

### 2.11. Statistical Evaluation

Statistical evaluation was performed using IBM SPSS statics software (version 25.0; IBM Japan, Tokyo, Japan). The results of the cell culture tests were analyzed by multiple comparisons using the Games–Howell and Tukey tests after one-way ANOVA. Moreover, microCT BMD images were analyzed using the Mann–Whitney U-test, the volume ratio of newly formed bone was analyzed by the t-test, and area ratio of newly formed bone based on histological examination was analyzed by Wilcoxon’s signed rank test because the data of area ratio of newly formed bone were not homoscedastic.

## 3. Results

### 3.1. Absorbance Analysis of Ca and P Concentrations in Supernatants

**Figure 3:**
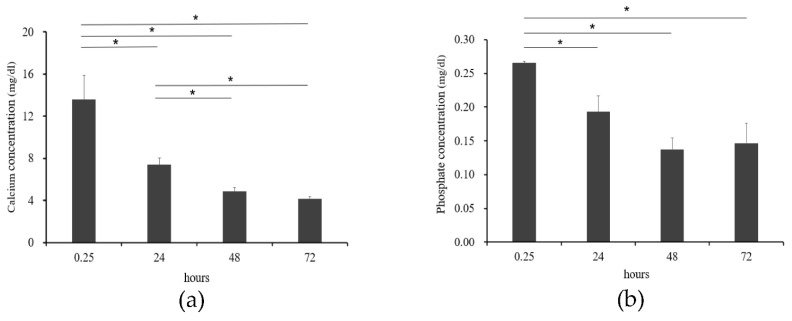
Changes in Ca (**a**) and P (**b**) concentration in the supernatant of supersaturated calcium phosphate solution (n = 4, * *p* < 0.05).

The Ca concentration in supernatants 15 min to 48 h after the preparation of the supersaturated calcium phosphate solution showed significant decreases with time but no significant difference between 48 and 72 h after the preparation (Figure 3a). Moreover, the P concentration decreased significantly with time from 15 min to 48 h after the preparation but showed no change after 48 h (Figure 3b).

### 3.2. Morphological Observation of Precipitates

**Figure 4:**
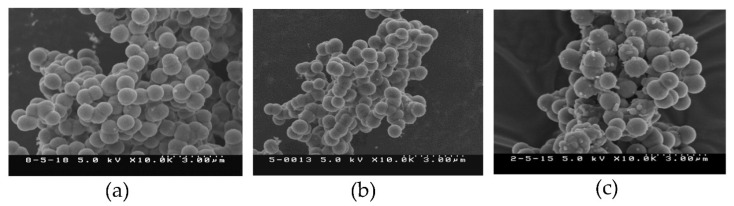
SEM images of precipitate: (**a**) after 24 h, (**b**) after 48 h, and (**c**) after 72 h.

The precipitates collected 24, 48, and 72 h after the preparation of supersaturated calcium phosphate became powdery when they were allowed to dry, and SEM images of the powder obtained 24 h after preparation of the solution showed clusters of spherically chained crystals (Figure 4a). However, no marked difference was observed in the size of the clusters after 48 or 72 h (Figure 4b,c).

### 3.3. XRD and FT-IR Analysis of Precipitates

**Figure 5:**
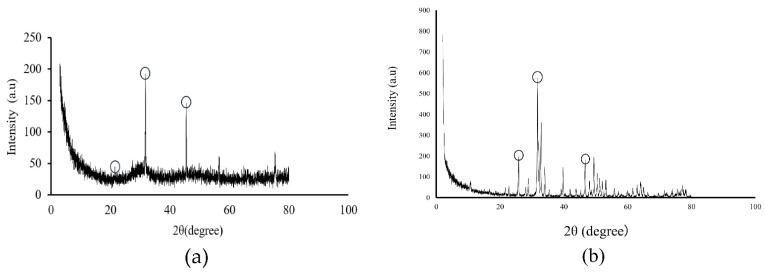
XRD analysis of (**a**) the peak of precipitate, (**b**) the peak of HA granules.

**Figure 6:**
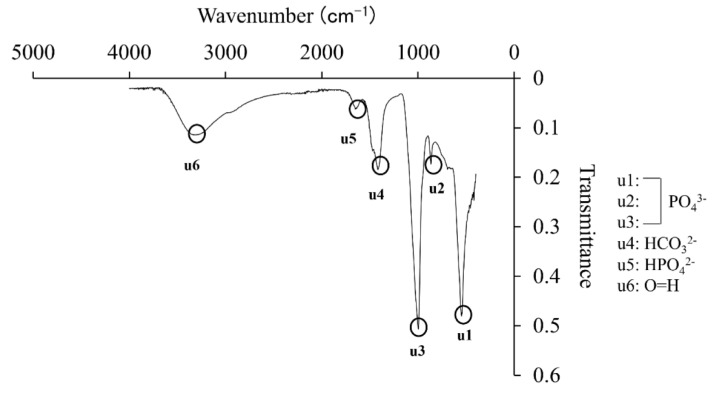
FT–IR analysis of precipitate.

XRD analysis showed peaks at 26°, 31° and 45°, and the intensity was generally low (Figure 5a). The peaks of the precipitate showed a similar peak peculiar to HA when compared with the HA granules (Figure 5b). In the IR spectrum of FT–IR, the peaks μ1, μ2, and μ3 were phosphates, μ4 at 1300 was carbonate radial, and μ5 at 1600 was hydrogen phosphate radical, and μ6 showed stretching vibration of OH that definitively identified HA (Figure 6).

### 3.4. Observation of the Surface of Biphasic HA Granules

**Figure 7:**
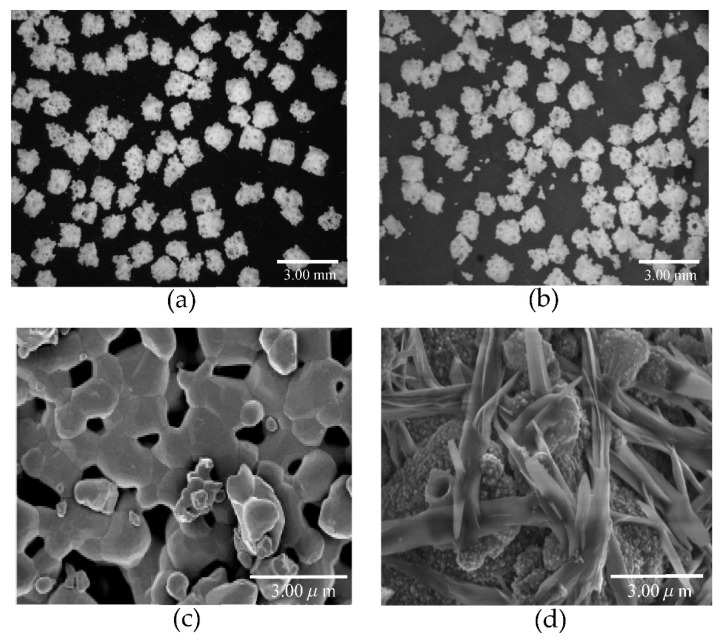
Stereo microscopic and SEM images of HA granules. (**a**): Stereo microscopic image of HA granules. (**b**): Stereo microscopic image of biphasic HA granules. (**c**): SEM image of HA granules. (**d**): SEM image of biphasic HA granules.

Figure 7 shows stereo microscopic and SEM images of biphasic HA granules prepared by immersing HA granules in the supersaturated calcium phosphate solution for 72 h.

In the stereo microscopic images, no difference was observed in the appearance between HA and biphasic HA granules (Figure 7a,b), but, in the SEM images, the surface of the HA granules was smooth but that of the biphasic HA granules was delicately coarse, and needle-shaped crystals varying in thickness grew and filled the spaces among the granules (Figure 7d).

### 3.5. Cytotoxicity Testing

**Figure 8:**
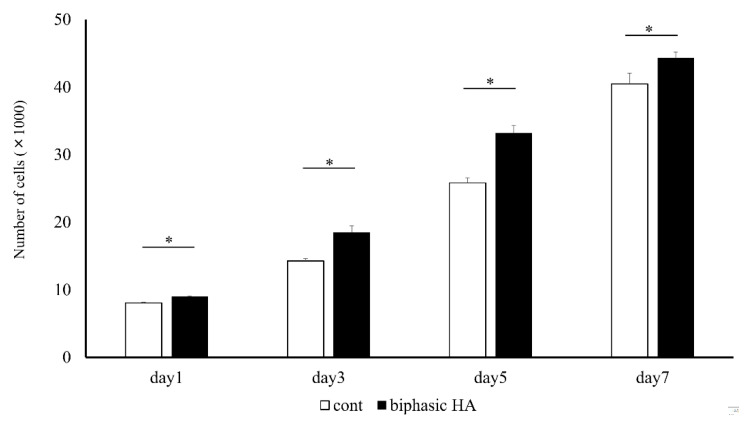
Cell proliferation ability of HA granules and biphasic HA granules (n = 4, * *p* < 0.05).

Since significant cell proliferation with time was observed in biphasic HA granules compared with the controls, low crystalline HA was confirmed to have no cytotoxicity (Figure 8).

### 3.6. Cell Differentiation Testing

**Figure 9:**
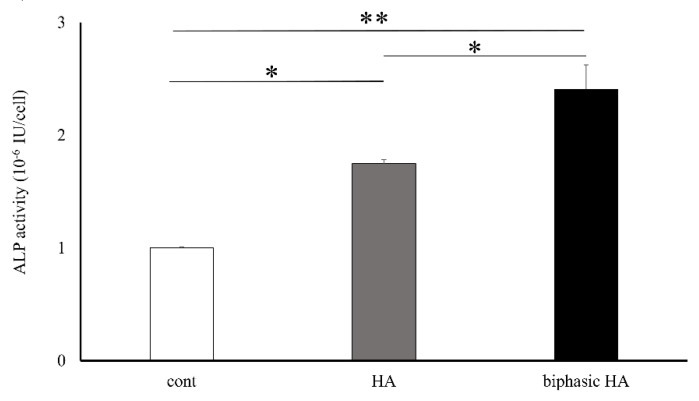
Bone differentiation ability of HA granules and biphasic HA granules (n = 4, * *p <* 0.05 ** *p <* 0.01).

The ALP activity seven days after the induction of bone differentiation was significantly higher in biphasic HA granules compared with the control and HA granules (Figure 9).

### 3.7. Animal Experiment

**Figure 10:**
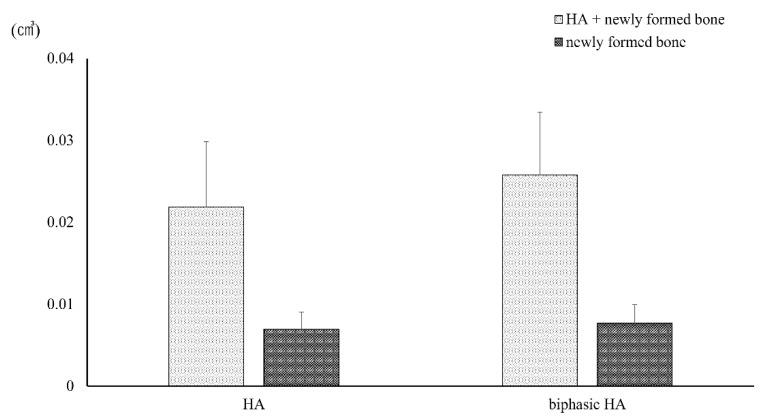
Volumes of HA granules and newly formed bone in ROI (n = 7).

**Figure 11:**
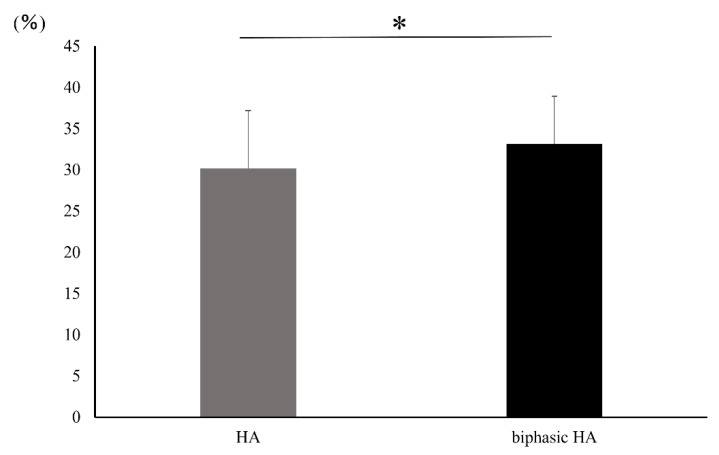
Volume ratio of newly formed bone in ROI (n = 7, * *p* < 0.05).

No significant difference was observed in the volumes of HA and newly formed bone or the volume of newly formed bone alone in ROI set in microCT images between the HA granule and biphasic HA granule groups (Figure 10). However, a significant difference was observed in the volume ratio of newly formed bone between the biphasic HA and HA groups (Figure 11).

**Figure 12:**
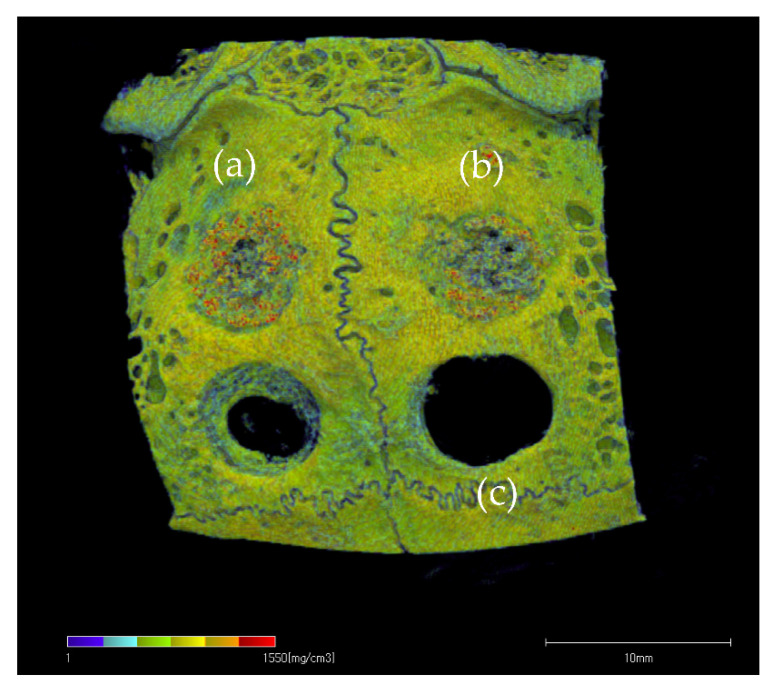
BMD image: (**a**) biphasic HA granules, (**b**) HA granules, (**c**) bone defect.

**Figure 13:**
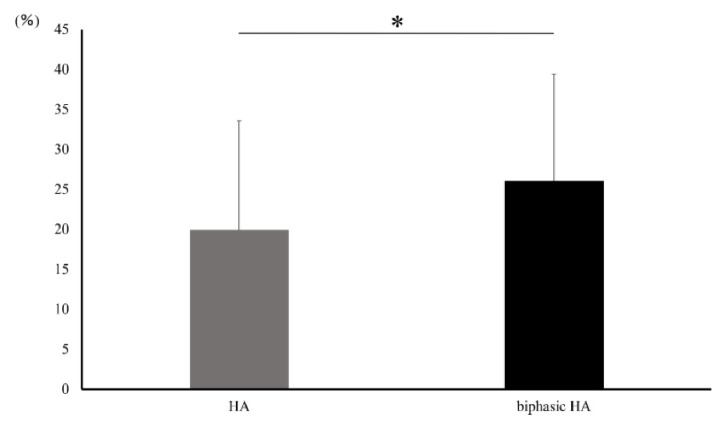
BMD in ROI in BMD images (n = 7, * *p <* 0.01).

**Figure 14:**
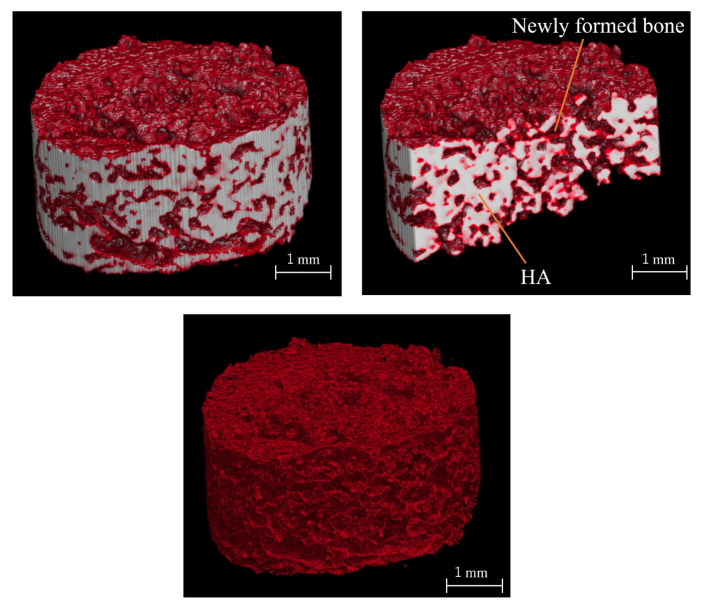
ROI extraction 3DCT images of bone defects filled with biphasic HA granules. (White: HA granules, Red: Newly formed bone).

Figure 12 shows the BMD images. In the unfilled negative control defects, only slight new bone formation was observed along the rim of existing bone, and the bone defects remained nearly unrepaired. In the defects filled with HA or biphasic HA, BMD similar to that of existing bone was restored in the entire bone defects. However, the area with high BMD was larger in the biphasic HA group and showed a significant difference compared with the HA group (Figure 13). Figure 14 shows ROI extraction 3DCT images of the area filled with biphasic HA. The white parts are HA, and the red parts are newly formed bone, and newly formed bone was found to be formed not only in the spaces among HA granules but also inside the pores. Moreover, in the selective 3DCT images of newly formed bone, new bone was formed evenly also in the spaces among HA granules.

**Figure 15:**
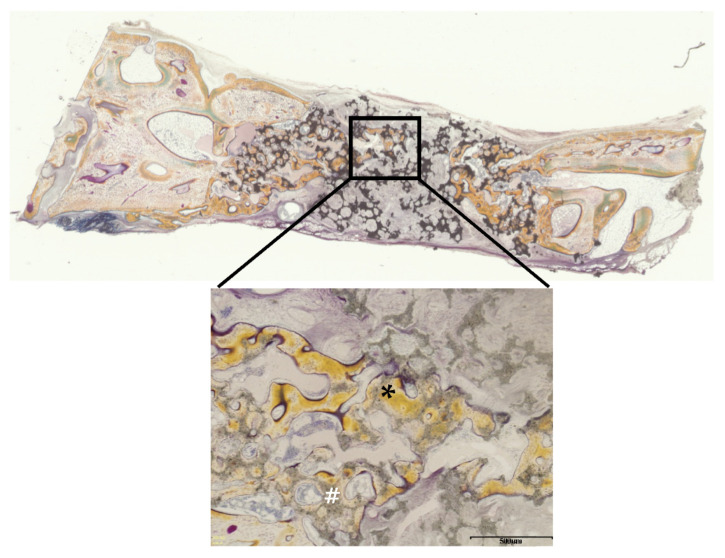
Villanueva bone stain images four weeks after filling with HA granules. (*: newly formed bone, #: HA granules, scale bar: 500 μm).

**Figure 16:**
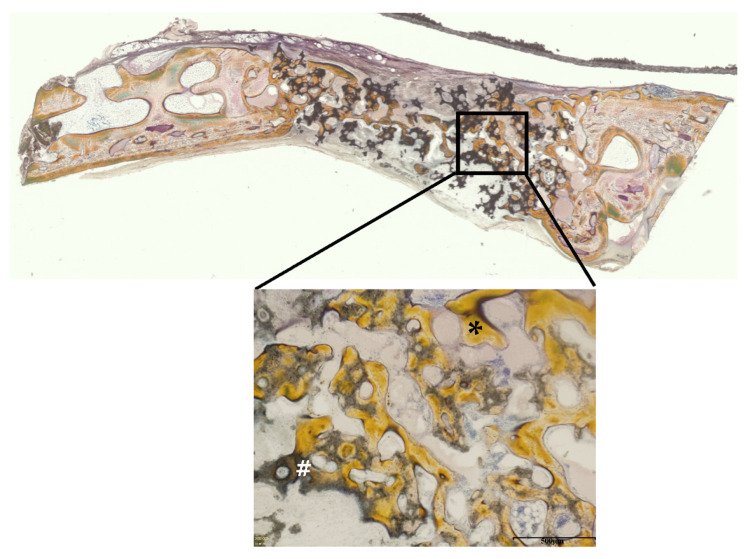
Villanueva bone stain images four weeks after filling of biphasic HA granules. (*: newly formed bone, #: HA granules, scale bar: 500 μm).

**Figure 17:**
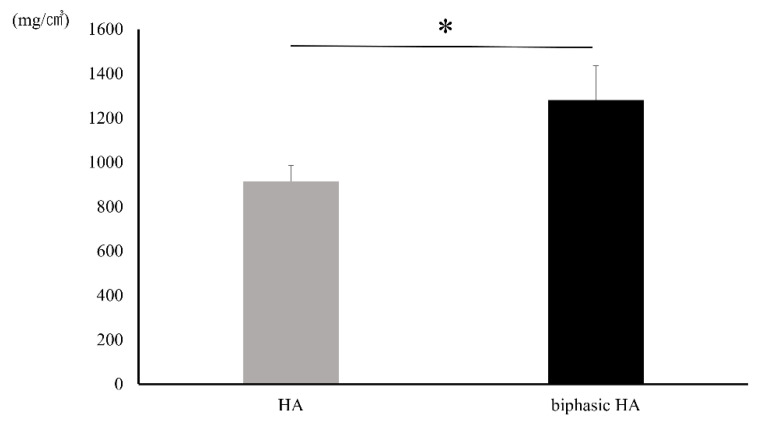
Area ratio of newly formed bone (n = 7, * *p* < 0.05).

Figure 15 and Figure 16 show Villanueva bone stain images of HA and biphasic HA granules four weeks after filling. New bone was formed from the margins of existing bone in both groups, thick trabeculae of newly formed bone were observed around biphasic HA granules, and the area ratio of newly formed bone was significantly higher compared with the HA granule group (Figure 17).

## 4. Discussion

Recently, the number of older patients who wish to have dental implant therapy is increasing. However, bone metabolism decreases with age, and implant therapy is often made difficult by resorption and atrophy of alveolar bone, so cases that require bone augmentation are increasing [1,2,3]. Bone substitutes are also used for bone augmentation in implant therapy, but as they have only osteoconductive property, they are inferior in the speed and amount of new bone formation compared with osteogenic and osteoinductive autologous bone grafts [4]. Therefore, there have been reports of promotion of osteogenesis by having bone substitutes support growth factors such as fibroblast growth factor-2 (FGF-2) and platelet-derived growth factor (PDGF) [21,22,23].

There have also been studies to promote new bone formation by having an apatite layer form on the surface of Bioactive glass or titanium using SBF [13], but no study of promoting new bone formation with an apatite layer precipitated on the surface of a bone substitute such as calcined bovine bone and synthetic apatite has been reported. Moreover, since the contents of Ca and P in SBF are nearly the same as those in human plasma, there was a limitation in new bone formation. Therefore, we directed our attention to the supersaturated calcium phosphate solution prepared by mixing medical infusion solutions [19] for more efficient precipitation of the apatite layer than bone substitute granules.

In this study, we first measured the Ca and P concentrations in the supernatants of the prepared supersaturated calcium phosphate solution. As a result, both the Ca and P concentrations decreased progressively until 48 h after the preparation but showed no change thereafter (Figure 4). This is considered to be explained by precipitation of Ca and P in the solution as crystals of calcium phosphate. When this precipitate was observed by SEM, clusters of spherically chained crystals were observed after 24 h (Figure 3). Therefore, this precipitate was suggested to be clusters of crystals of calcium phosphate. Moreover, since no change was observed in the concentrations of Ca and P or the size of clusters more than 48 h after preparation of the supersaturated calcium phosphate solution, the precipitation of calcium phosphate crystals is considered to have nearly ended within 24 h after the preparation of supersaturated calcium phosphate solution.

Moreover, when this precipitate was analyzed by XRD analysis to identify it, peaks characteristic of HA were observed at 26°, 31° and 45°, and the intensity of diffraction was generally low (Figure 5). In addition, FT–IR spectroscopy showed an absorption spectrum with phosphate ion, hydrogen phosphate ion, carbonate ion, and a stretching vibration of OH, which definitively identify HA (Figure 6). Therefore, the precipitate from the supersaturated calcium phosphate solution prepared by mixing five medical infusion solutions was shown to be low crystalline HA [24,25]. HA is classified as nonbioabsorbable high-temperature HA prepared by adjusting the Ca/P ratio at 1.67 and sintering it at a high temperature of ≥1200 °C and cooling it rapidly, and low-temperature HA generated spontaneously in a natural environment at ambient temperature, room temperature, or body temperature or in the body [26,27]. Since low crystalline HA is bioabsorbable, has high biological activities, and has excellent osteoconductivity, it is expected to promote new bone formation more than high-temperature HA [28].

Next, we prepared biphasic HA granules with low crystalline HA precipitated on the surface of HA granules by immersing them for 72 h in a supersaturated calcium phosphate solution. When they were examined under the stereo microscopy and SEM, marked growth of thick needle-shaped low crystalline HA was observed (Figure 7d). Ca and P ions in SBF or supersaturated calcium phosphate solution are considered to form Ca/P clusters, which become amorphous calcium phosphate (ACP) on the surface of HA granules, precipitate as low crystalline HA as ACP bind together, and grow to form needle-shaped crystals [28]. We evaluated how this low crystalline HA affects cells with osteogenic properties in vitro.

It has been reported that MC3T3-E1 cells derived from the mouse cranium used in an in vitro study differentiate into osteoblasts and play a role in osteogenesis [29]. In a proliferation test of these MC3T3-E1 cells, biphasic HA granules showed a significantly higher cell proliferation ability than HA granules. This indicated that low crystalline HA has no cytotoxicity (Figure 8). Next, we evaluated the effects of low crystalline HA on bone differentiation. As a result, biphasic HA granules showed a significantly higher bone differentiation ability compared with the control and HA granules (Figure 9). Ca and P ions promote bone regeneration by regulating the activities of osteoblasts and osteoclasts. In addition, the surface properties and porosity of calcium phosphate affect cell growth [30]. Moreover, since ACP, among calcium phosphate ceramics, is more soluble than HA, it has more rapid osteoinductivity and promotes osteogenesis. Ca ion released from the surface of ACP is reported to activate cell signal transmission via ion channels of MC3T3E-1 cells and promote osteoblast differentiation [31]. Therefore, low crystalline HA that showed needle-like growth on the surface of HA granules by precipitation of Ca and P in the supersaturated calcium phosphate solution was considered to have biological activity to promote osteogenesis. Moreover, as the smooth surface of HA granules changed to a coarse surface with needle-like crystals, the surface area was considered to have increased to facilitate the adhesion and proliferation of cells. For these reasons, we decided to evaluate the promotion of new bone formation in vivo using biphasic HA granules.

In this animal experiment, full-thickness bone defects 6.0 mm in diameter, which are considered to be critical bone defects, were created in the rabbit cranium and were filled with biphasic HA granules and HA granules as the control. As a result, marked new bone formation from existing bone was observed around both biphasic HA and HA granules four weeks after filling, but BMD was significantly higher in the biphasic HA group, and extracted 3DCT of ROI showed new bone formation even inside pores. Moreover, when the amount of new bone formation was compared in microCT images, no significant difference was observed between the two groups, but the volume ratio of new bone was significantly higher in the biphasic HA group. Moreover, histological examination showed thick trabeculae of new bone formed around biphasic HA granules and a significant difference in the area ratio of newly formed bone. Low crystalline HA is dissolved by H^+^ ion supplied by the proton pump of osteoclasts and is involved in new bone formation by transmitting the signal to osteoblasts [30] Therefore, HA collagen compounded sponge prepared by binding nanosized low crystalline HA to type I collagen fibers has been reported to have induced significantly higher new bone formation in the rabbit femur compared with HA granules or βTCP granules after four weeks due to resorption by osteoclasts two weeks after its grafting and subsequent new bone formation [32]. Moreover, the needle-like crystals on the surface of biphasic HA granules were considered to have served as a scaffold appropriate for adhesion, proliferation, and differentiation of cells related to osteogenesis, and thus, enhanced osteoconductivity and promoted new bone formation.

In this study, biphasic HA granules prepared by precipitating low crystalline HA obtained by mixing five medical infusion solutions on the surface of HA granules were shown to have biological activities to enhance osteoconductivity.

## 5. Conclusions

Biphasic HA granules prepared by compounding HA granules with low crystalline HA precipitated by mixing five medical infusion solutions were shown to promote new bone formation. Therefore, biphasic HA granules are suggested as a bone substitute useful for bone augmentation in implant therapy.

## Figures and Tables

**Table 1:** Compositions of medical infusion preparations used for the preparation of supersaturated calcium phosphate solution.

Solution Composition	Calcium-Containing Solution (mM)		Phosphorus-Containing Solution (mM)		NaHCO_3_ Solution (mM)	Calcium-Phosphate Solution (mM)
	Ringer’s Solution	Concyte-Ca	Klinisalz B	Conclyte-P	Meylon	-
pH (37 °C)	-	-	-	-	-	7.82
Volume (mL)	8.135	0.037	0.898	0.019	0.911	10.00
Na^+^	147.00	-	45.0	-	833.00	-
K^+^	4.00	-	25.0	1000.00	-	7.41
Mg^2+^	-	-	5.00	-	-	-
Ca^2+^	4.5	500.00	-	-	-	7.54
Cl^−^	156.00	1000.00	45.00	-	-	-
HP_2_O_4_^2−^	-	-	10.00	-	-	-
HPO_4_^2−^	-	-	-	500.00	-	-
HCO_3_^−^	-	-	-	-	833.00	-
CH_3_COO^−^	-	-	20.00	-	-	-

## Data Availability

Not applicable.

## References

[1] Chavda S., Levin L. (2018). Human studies of vertical and horizontal alveolar ridge augmentation comparing different types of bone graft materials: A systematic review. J. Oral Implantol..

[2] Kudoh K., Fukuda N., Kasugai S., Tachikawa N., Koyano K., Matsushita Y., Ogino Y., Ishikawa K., Miyamoto Y. (2019). Maxillary sinus floor augmentation using low-crystalline carbonate apatite granules with simultaneous implant installation: First-in-human clinical trial. J. Oral Maxillofac. Surg..

[3] Nakagawa T., Kudoh K., Fukuda N., Kasugai S., Tahikawa N., Koyano K., Matsushita Y., Sasaki M., Ishikawa K., Miyamoto Y. (2019). Application of low-crystalline carbonate apatite granules in 2-stage sinus floor augmentation: A prospective clinical trial and histomorphometric evaluation. J. Periodontal Implant Sci..

[4] Matsuno T., Omata K., Hashimoto Y., Satoh T. (2010). Alveolar bone tissue engineering using composite scaffolds for drug delivery. Jpn. Dent. Sci. Rev..

[5] Shigeishi H., Takechi M., Nishimura M., Takamoto M., Minami M., Ohta K., Kamata N. (2012). Clinical evaluation of novel interconnected porous hydroxyapatite ceramics (IP-CHA) in a maxillary sinus floor augmentation procedure. Dent. Mater. J..

[6] Cabezas-Mojón J., Barona-Dorado C., Gómez-Moreno G., Fernández-Cáliz F., Martínez-González J.M. (2012). Meta-analytic study of implant survival following sinus augmentation. Med. Oral Patol. Oral Cir. Buccal.

[7] Rickert D., Slater J.J.R.H., Meijer H.J.A., Vissink A., Raghoebar G.M. (2012). Maxillary sinus lift with solely autogenous bone compared to a combination of autogenous bone and growth factors or (solely) bone substitutes. A systematic review. Int. J. Oral Maxillofac. Surg..

[8] Draenert F.G., Huetzen D., Neff A., Mueller W.E.G. (2014). Vertical bone augmentation procedures: Basics and techniques in dental implantology. J. Biomed. Mater. Res. A.

[9] Suwimon B., Eileen G., Raffaella C., Nicholas D.E., David W.M., Alexandra E.P., Molly M.S. (2012). The role of intracellular calcium phosphate in osteoblast-mediated bone apatite formation. Proc. Natl. Acad. Sci. USA.

[10] White A.A., Best S.M., Kinloch I.A. (2007). Hydroxyapatite-carbon nanotube composites for biomedical applications: A review. Int. J. Appl. Ceram. Technol..

[11] Agnieszka S.K., Anna D., Wioletta F., Magdalena G., Sonia K.K., Dagmara S., Agnieszka T., Bozena T. (2021). Review of the applications of biomedical compositions containing hydroxyapatite and collagen modified by bioactive components. Materials.

[12] Kokubo T., Takadama H. (2006). How useful SBF in predicting in vivo bone bioactivity?. Biomaterials.

[13] Kokubo T., Ito S., Shigematsu M., Sanka S., Yamamuro T. (1987). Fatigue and life-time of bioactive glass-ceramic A-W containing apatite and wollastonite. J. Mater. Sci..

[14] Kokubo T., Kushitani H., Sakka S., Kitsugi T., Yamamuro T. (1990). Solutions able to reproduce in vivo surface-structure change in bioactive glass-ceramic A–W^3^. J. Biomed. Mater. Res..

[15] Kokubo T., Ito S., Huang Z.T., Hayashi T., Sakka S., Kitsuji T., Yamamuro T. (1990). Ca, P-rich layer formed on high-strength bioactive glass-ceramic A-W^3^. J. Biomed. Mater. Res..

[16] Filgueiras M.R., Torre G.L., Hench L.L. (1993). Solution effects on the surface reactions of a bioactive glass. J. Biomed. Mater. Res..

[17] Ohtsuki C., Kushitani H., Kokubo T., Kotani S., Yamamuro T. (1991). Apatite formation on the surface of Ceravital-type glass-ceramic in the body. J. Biomed. Mater. Res..

[18] Oyane A., Uchida M., Choong C., Triffitt J., Jones J., Ito A. (2005). Simple surface odification of poly (ε-caprolactone) for apatite deposition from simulated body fluid. Biomaterials.

[19] Mutsuzaki H., Ito A., Sakane M., Sogo Y., Oyane A., Ebihara Y., Ichinose N., Ochiai N. (2007). Calcium phosphate coating formed in infusion fluid mixture to enhance fixation strength of titanium screws. J. Mater. Sci..

[20] Yanagisawa Y., Ito A., Hara Y., Mutsuzaki H., Murai S., Fujii K., Sogo Y., Hirose M., Oyane A., Kobayashi F. (2018). Initial clinical trial ofpins coated with fibroblast growth factor-2-apatite composite layer in external fixation of distal radius fractures. J. Orthop..

[21] Mutsuzaki H., Ito A., Sakane M., Sogo Y., Oyane A., Ochiai N. (2008). Fibroblast growth factor-2-apatite composite layers on titanium screws to reduce pin tract infection rate. J. Biomed. Mater. Res. B Appl. Biomater..

[22] Fujii K., Ito A., Mutsuzaki H., Murai S., Sogo Y., Hara Y., Yamazaki M. (2017). Reducing the risk of impaired bone apposition to titanium screws with the use of fibroblast growth factor-2-apatite composite layer coating. J. Orthop. Surg. Res..

[23] Davies O.G., Grover L.M., Lewis M.P., Liu Y. (2018). PDGF is a potent inhibitor of bone formation in a tissue engineered model of pathological ossification. J. Tissue Eng. Regen. Med..

[24] Sharma K., Sharma S., Thapa S., Bhagat M., Kumar V., Sharma V. (2020). Nanohydroxyapatite-, gelatin-, and acrylic acid-based novel dental restorative material. ACS Omega.

[25] Dagmara M., Kamila B., Agnieszka S.K. (2013). Studies on sintering process of synthetic hydroxyapatite. Acta Biochim. Pol..

[26] Shimada Y., Chow L.C., Takagi S., Tagami J. (2010). Properties of Injectable Apatite- Forming Premixed Cements. J. Res. Natl Stand. Technol..

[27] Kikuchi M., Itoh S., Ichinose S., Shinomiya K., Tanaka J. (2001). Self-organization mechanism in a bone-like hydroxyapatite/collagen nanocomposite synthesized in vitro and its biological reaction in vivo. Biomaterials.

[28] Dey A., Bomans P.H., Müller F.A., Will J., Frederik P.M., de With G., Sommerdijk N.A. (2010). The role of prenucleation clusters in surface-induced calcium phosphate crystallization. Nat. Materials..

[29] Nakajima T. (1991). Effects of hypergravity on Migration, Proliferation and Function of Mouse Osteoblastic Cell Line MC3T3-E1. J. Stomatol. Soc. Jpn..

[30] Jiwoon J., Jun H.K., Jung H.S., Nathaniel S.H., Chan Y.H. (2019). Bioactive calcium phosphate materials and applications in bone regeneration. Biomater. Res..

[31] Satyavrata S., Abby R.W., Aaron S.G. (2013). Calcium phosphate ceramics in bone tissue engineering: A review of properties and their influence on cell behavior. Acta Biomater..

[32] Tsuchiya A., Sotome S., Asou Y., Kikuchi M., Koyama Y., Ogawa T., Tanaka J., Shinomiya K. (2008). Effects of pore size and implant volume of porous hydroxyapatite/collagen (Hap/Col) on bone formation in a rabbit bone formation in a rabbit bone defect model. J. Med. Dent. Sci..

